# Effect of Cu, Ni and Pb doping on the photo-electrochemical activity of ZnO thin films

**DOI:** 10.1039/c8ra10599e

**Published:** 2019-03-07

**Authors:** Ahmed A. Aboud, Mohamed Shaban, Neerish Revaprasadu

**Affiliations:** Department of Physics, Faculty of Science, Beni-Suef University Beni-Suef 62514 Egypt ahmed.mostafa@science.bsu.edu.eg; Department of Chemistry, University of Zululand Private Bag X1001 Kwa-Dlangezwa 3886 South Africa

## Abstract

In the present study, the effects of metallic doping on the photoelectron\chemical properties of zinc oxide thin films have been studied. All films have been deposited using the spray pyrolysis technique at a constant doping level of 3 wt% whereby Cu, Ni, and Pb were used as dopants. The structure of all films was studied by X-ray diffraction which showed the grain size of all doped films to be 50 nm. The energy band gap of all films was estimated using optical transmission spectroscopy. The Ni, Cu, and Pb-doped ZnO photoelectrodes were applied for the photoelectrochemical (PEC) H_2_ generation from H_2_O. Pb doping leads to the highest photocurrent of the ZnO photoelectrodes. The current density–potential characteristics were measured under white light and monochromatic illumination. The stability of the electrode was quantified as a function of the number of H_2_ production runs and exposure time. Finally, the incident photon-to-current conversion efficiency, IPCE, and applied bias photon-to-current efficiency, ABPE, were calculated. The optimum IPCE at 390 nm was ∼30% whereas the ABPE was 0.636 at 0.5 V.

## Abstract 2

In the present study, the effects of metallic doping on the photoelectron\chemical properties of zinc oxide thin films have been studied.

## Graphical abstract

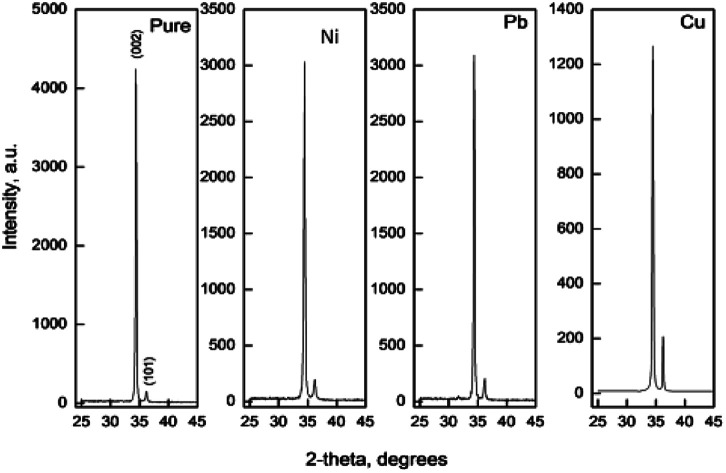

## 1. Introduction

The depletion of fossil fuel resources has contributed towards a renewed interest in the field of energy generation and storage. Photochemical water splitting is a popular area of interest since water covers 71% of the earth's surface. Transition metal oxides are potential candidates for photoelectrochemical (PEC) applications to generate hydrogen from water.^1–3^ Despite the fact that wide bandgap semiconductors are comparatively more stable than their small band gap^4^ counterparts, their application in PEC cells is limited as they can absorb only high energy photons (in the UV region) which hardly constitute 3% of solar radiation.^4^ The principle drawbacks in the use of metal oxide films for water-splitting are the large band gaps, the absence or lack of visible light absorption from sunlight illumination, the limited charge transport due to the lack of continual conducting paths in 0D nanoparticulate films, and the loss of photons due to surface reflection.^5^ As a result, a variety of 1D nanostructures have been introduced to improve the charge-transportation and to reduce surface reflection effects.^6^ Also, the coupling between narrow band-gap semiconductors (CdS, CdTe, CdSe, CuInS_2_) and wide band-gap semiconductors (TiO_2_, ZnO) was suggested to enhance visible light absorption and hence PEC efficiencies for efficient hydrogen production.^7,8^ Previously, many wide/narrow band-gap multicomponent semiconductors with different and morphologies have been studied.^9–12^

ZnO is a wide bandgap n-type semiconductor with a band gap value of 3.2 eV with an anisotropic crystal structure.^13,14^ It has been extensively studied and its properties well reported.^4,15,16^ Also, non-toxicity, abundance in nature, low-cost and easy synthesis, massive production, and high thermal stability are advantageous properties of ZnO.^17,18^ Moreover, the morphologies of ZnO can be tuned to produce a large variety of superstructures with different morphologies and orientations due to it's hexagonal (wurtzite) crystal.^13,19,20^

The PEC water splitting is a complex thermodynamic process that required the participation of two water molecules in two steps to split the atoms in H_2_O. The products are separated based on the reduction half-reaction [2H^+^ + 2e^−^ → (*E*_red_ = 0.00 V/RHE)] and oxidation half-reaction [2H_2_O + 4h^+^ → O_2_ + 4H^+^ (*E*_oxi_ = −1.23 V/RHE)]. The overall PEC reaction can be represented as 2H_2_O → 2H_2_ + O_2_ (Δ*E* = −1.23 V/RHE).^21^ Since H_2_O oxidation is the rate-limiting reaction in the overall PEC water splitting, the design of an efficient photoanode is an important barrier to overcome to perform this reaction.^22^

For efficient PEC water splitting, ZnO is a prominent semiconductor owing to its low cost and high catalytic efficiency. ZnO nanostructures have shown interesting results in PEC water splitting because of their outstanding electrochemical properties.^23^ Rokade *et al.*^24^ designed 1D ZnO nanorods and nanotubes (NTs) by the electrodeposition method, the nanotube structure showed enhanced hydrogen PEC current density of 0.67 A cm^−2^ at 0.5 V over the nanorod morphology.

In addition, M. Liu *et al.*^25^ investigated the water splitting and chemical stability of ZnO/titania core/shell nanowire photoanodes, which showed 25% higher PEC water splitting activity than the as-deposited ZnO. Zhang *et al.* showed that the design of high-quality ZnO films with limited deep trap states and thickness greater than the light absorption depth and space charge region width provided a long photocarrier lifetime with a high quantum efficiency (>80%).^26^

Also, Hoang *et al.*^27^ fabricated an arrayed structure of CdS-sensitized ZnO nanorods on ITO substrates for PEC hydrogen generation. They got maximum photoconversion efficiency (2.7%) under solar irradiation of 100 mW cm^−2^ for PEC hydrogen generation using the electrode grown for 3 h. The maximum hydrogen rate was 22 mL cm^−2^ after 1 h exposure. Moreover, Bouhjar *et al.*^21,28^ showed that the PEC efficiency of the hydrothermally grown α-Fe_2_O_3_/ZnO heterojunction is higher than that of FTO/α-Fe_2_O_3_ and FTO/ZnO. Furthermore, Kim *et al.*^29^ introduced complex co-sensitized ZnO/WO_*x*_ heterostructures for efficient visible light absorption and PEC hydrogen production.

The CdSe/CdS co-sensitized ZnO/WO_*x*_ showed effective charge transport and produced a high PEC current density of 11 mA cm^−2^ at 0.5 V. Desai *et al.*^30^ recently discussed different modern approaches for producing different morphologies of ZnO superstructures and highlighted their role in improving PEC performance towards the efficient production of hydrogen.

Appavoo *et al.*^31^ used *in situ* ultrafast spectroscopy to quantify the recombination processes (bulk and surface) in nanostructured photocatalysts for PEC water splitting and showed how these recombination processes limit the overall efficiency. Moreover, Cen *et al.*^32^ showed that different *in situ* spectroscopic and microscopic methods can be combined to develop mechanistic information about all features of PEC-WS.

The application of ZnO and ZnO-based structures in solar water splitting is still limited due to its high band gap energy, poor stability in aqueous media, insufficient photo-conversion efficiency, charge transfer resistance, or complicated architecture.^33–36^ Therefore, it is meaningful to address a low-cost and straightforward strategy to introduce different metal doping to 1D ZnO nanostructured films. The metal-doped ZnO with high charge carrier density (electrical conductivity) and enhanced light absorption is introduced to improve PEC performance. Also, in order to utilize such materials in PEC applications, the band gap value has to be reduced. N-doping has been used to shrink the ZnO band gap.^15,16,37^ Sundhakar *et al.*^16^ studied the effect of Al and N co-doping on the PEC activity of r.f. magnetron sputtered ZnO thin films on FTO glass without addressing stability and evaluation of PEC performance. Moreover, compared with other methods such as r.f. magnetron sputtering, chemical vapour deposition (CVD), and plasma chemical vapour deposition (PCVD). The spray pyrolysis technique has been known to be highly cost-effective and adaptable for the deposition of doped ZnO.

In this paper, we report the deposition of pure, Cu, Ni and Pb-doped zinc oxide thin films by the spray pyrolysis technique. All films have been obtained using the same deposition parameters except the type of the dopant. The structural, optical, and PEC properties are studied and discussed. Finally, electrode stability and PEC performance are evaluated under monochromatic and white light illumination to elucidate the effect of doping.

## 2. Experimental

An amount of 7.0 mg zinc acetate dihydrate was dissolved in 60.0 mL of ethanol at room temperature and stirred for 15 min till the solution becomes transparent. In the case of doping (3 wt%) with Pb, Ni and Cu a calculated amount of lead acetate, nickel acetate and copper chloride was dissolved in ethanol and added to the precursor solution. Microscope glass slides (2.0–2.5 cm^2^) were used as substrates which were cleaned in mineral acid (sulphuric acid and nitric acid), in alcohols (methanol and ethanol) and then dried under air stream. During the spray process, the temperature of the substrate was kept constant at 450 °C, and the air flow rate was 25 mL min^−1^. The distance between the spray nozzle and the substrate was 36 cm. The deposited films were characterized by X-ray diffraction with Cu Kα radiation (*λ* = 1.54 Å) on a DX-2500 diffractometer. The optical transmittance spectra were recorded in the spectral range of 200–1100 nm.

Ni, Cu, and Pb-doped ZnO electrodes were used as working electrodes for photocatalytic hydrogen generation water splitting experiments. The PEC behaviour was measured under illumination of 500 W Mercury-Xenon Light Source (Newport, 66926-500HX-R07) provided with a series of linear optical filters in 0.3 M (100 mL) Na_2_S_2_O_3_ solution at room temperature (25 °C) with a sweep rate of 1 mV s^−1^. The schematic diagram of the used PEC cell is shown in Fig. 5. The area of the used electrode is 1 cm^2^, the counter electrode being Pt-electrode, and the reference electrode, Ag/AgCl. The PEC current density–potential (*J*_ph_–*E*) and the current density–time (*J*_ph_–*t*) behaviours were recorded with a Keithley measurement source unit using LabTracer software (2400 Source Meter, A Tektronix Company).

## 3. Results and discussion

**Fig. 1:**
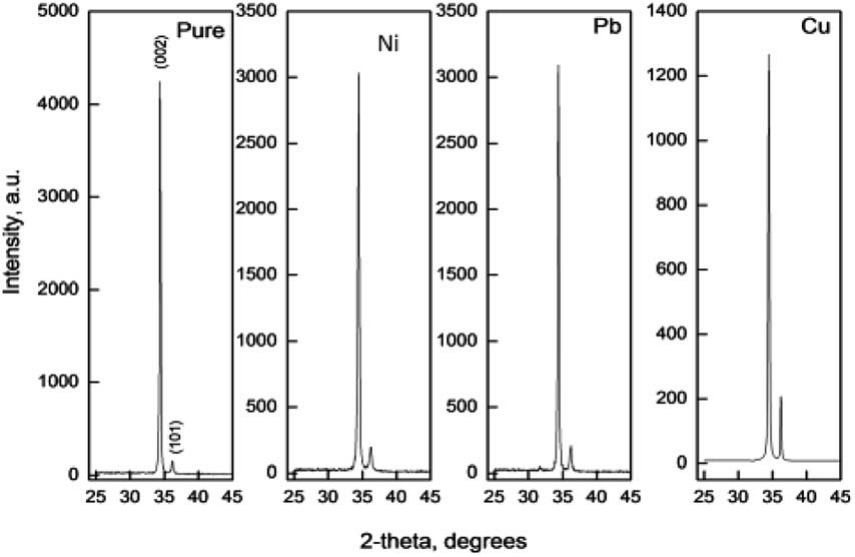
X-ray diffraction patterns for pure and Ni, Cu and Pb doped zinc oxide films.

Fig. 1 shows the main X-ray diffraction peaks for thin films of pure and doped ZnO films as a function in the dopant type. All films are found to be crystalline with the principle peak around 2*θ* = 34.4°. This peak matches well with the zinc oxide hexagonal ZnO ICDD card number (79-0205) which is attributed to the (002) plane.^9,10^ Another small peak, recorded at approx. 2*θ* = 36.2° is attributed to the (101) plane for the same ZnO phase. This reveals that the film had high preferential *c*-axis orientation. Generally, the intensity of the diffraction peaks decreases greatly with the increase of doping concentration, indicating a loss of crystallinity due to lattice distortion. When dopant ions are incorporated into the periodic crystal lattice of ZnO_2_, a strain is induced into the system, resulting in the alteration of the lattice periodicity and decrease in crystal symmetry. As can be seen from the XRD patterns, the diffraction peaks get broadened upon doping, suggesting a decrease in the grain size as listed in Table 1.

**Table 1:** Lattice parameters, grain size (GS), strain, stress, bond lengths, thickness and band gap

Film	*a* (Å)	*c* (Å)	GS (nm)	Strain	Stress	*u*	*L*	Thickness (nm)	Band gap (eV)
Pure	3.259596	5.21282	65	0.001117726	−260430190.1	0.380335	1.885729	753	3.2
Cu	3.250922	5.2028	50	−0.00080661	187939312.5	0.380142	1.880744	535	3.2
Ni	3.257866	5.21392	50	0.00132898	−309652391	0.380142	1.884745	482	3.2
Pb	3.25681	5.21608	50	0.001743806	−406306894.6	0.37995	1.884148	600	3.3

In all films, no other crystalline phases could be detected confirming the singularity of the hexagonal ZnO phase and the successful doping process. As observed, the main peak intensity decreases as a function of the dopant type. This could be attributed to the close ionic radii values between Zn and all dopants except Pb (Pb^4+^ = 0.78 Å (ref. 38) and Ni^+2^ = 0.69 Å,^39^ Cu^+2^ = 0.73 Å, Cu^+^ = 0.77 Å,^40^ Zn^+2^ = 0.74 Å (ref. 41)).

The grain size was calculated depending on the full width at half maximum (FWHM). The FWHM was found to increase upon doping. Such an increase in the FWHM value reflects the increase in the imperfections due to the increase in the internal microstrain within the film accompanied with a reduction in the grain size value. The grain size was estimated according to Scherrer's equation:^42^-
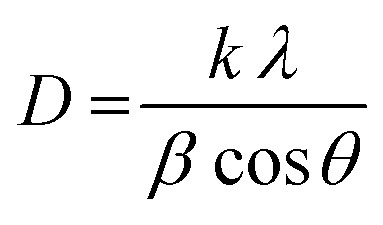
where *D* is the particle size, is the X-ray wavelength (1.54 Å), is the full width at half maximum in radian and is the angle value in radian. The obtained values are listed in Table 1. All doped films have the same value of the grain size of 50 nm which is lower than the grain size (65 nm) of pure zinc oxide films. The reduction in the grain size could be attributed to the formation of new nucleating centres caused by the reduction in the nucleation energy barrier.^43^

The influence of Cu-doping on the crystallization quality of ZnO thin films is attributed to the radius of Cu^2+^ (0.73 Å) bring less than that of Zn^2+^ (0.74 Å). When Cu^2+^ was incorporated in the ZnO lattice, the structure around Cu is deformed in small areas and the lattice constants decreases, see Table 1. Wang *et al.*^44^ observed the reduction of the (002) peak intensity for Cu-doped ZnO thin films prepared using the sputtering technique. This reduction in the peak intensity has been attributed to Cu-atoms which exist as interstitial instead of substitution positions where Cu^+^ and Cu^2+^ can be detected by XPS.

Doping of ZnO films with Ni^+2^ ions does not have appreciable effect on the crystal structure of the films as recorded by Reddy *et al.*^47^ for films deposited using the spray pyrolysis technique. But in the work of Patil *et al.*^48^ the grain size was found to increase first when the Ni content is lower than 1.5%. After this percentage the grain size was found to decrease with decrease in intensity of the (002) diffraction peak. Abdel-Wahab *et al.*^49^ recorded a crystallinity degradation of ZnO thin films upon Ni-doping. At Ni 7 wt% concentration, the (002) disappears. From the variations and disappearing of the peaks, it can be assumed that the doping of Ni modifies the film growth. Similar results can be observed by Nunes *et al.*^50^ and El Manouni *et al.*^51^ Also the degradation of the grain size upon Ni doping has been reported previously by Kim *et al.*^52^ İskenderoğlu *et al.*^46^ studied the impact of Ni-doping on the properties of ZnO thin films prepared by the spray pyrolysis technique. They found that the grain size decreased upon doping up to Ni-concentration of 6%. Thereafter the grain size starts to grow again.

Turgut *et al.*^53^ studied the effect of Pb doping on the properties of ZnO thin films deposited using sol–gel technique. They found that the (002) peak intensity decreases with Pb incorporation at high level where the films shows an amorphous nature at doping level 30 wt%.

Generally, the reduction in the grain size can be attributed in the frame of Zener pining effect with dopant acting as obstacle to inhibit grain growth. The limiting grain size, *D*, is generally given as a function of the mean radius of the pinning particles, *R*, and their volume of fraction, *f*_v_;^45^
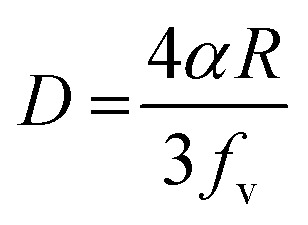
where *α* is a geometrical constant. The Zener drag, *Z*, is given by;
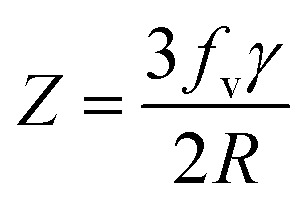
and where *γ* is the grain boundary energy so;
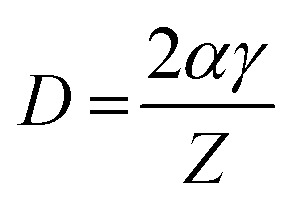
when *R* is a constant and *D* depends on 1/*f*_v_. Accordingly the high the dopant fraction the lower the *D* value.

The strain (*ε*) and stress (*σ*) in the film, along the *c*-axis, were estimated using the following equations:^54^-
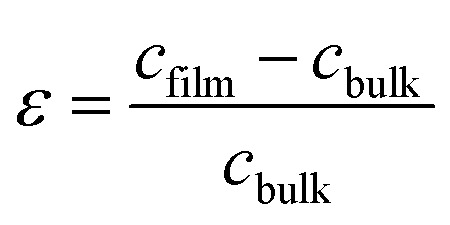
where *c*_film_ and *c*_bulk_ are the lattice parameters of the film and unstrained ZnO, respectively. Since the ionic radii of Pb is greater than that for Zn, Pb substituting Zn in the ZnO lattice may be the cause of tensile strain along with expansion of crystallite size. On the other hand the ionic radius of Cu^+2^ is smaller than that for Zn^+2^, Cu substituting Zn may result in compressive strain along with the reduction of crystallite size.

Zhong *et al.*^39^ studied the effect of Ni-doping on the properties of ZnO thin films where a compressive stress is found due to the substitution of Zn^2+^ (0.74 nm) with Ni^2+^ (0.69 nm). This result is in contradiction to ours where a tensile stress is found. That may suggest an interstitial doping of a small amount of Ni-ions in the network of ZnO.

The obtained values indicate the presence of a compressive strain in the case of Cu-doping and tensile strain is observed in the case of pure, Ni and Pb doped films. The lattice parameters of all films have been estimated from the equation:^54^-
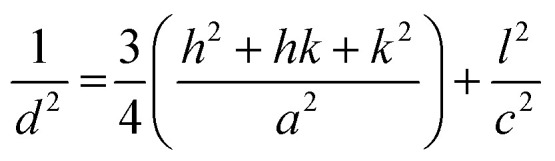


The variation in the lattice parameters values are listed in Table 1, *a* and *c*. This behavior could be attributed to the ionic radii value as mentioned above.

The Zn–O bond length (*L*) was also estimated using the following equations:^54^-
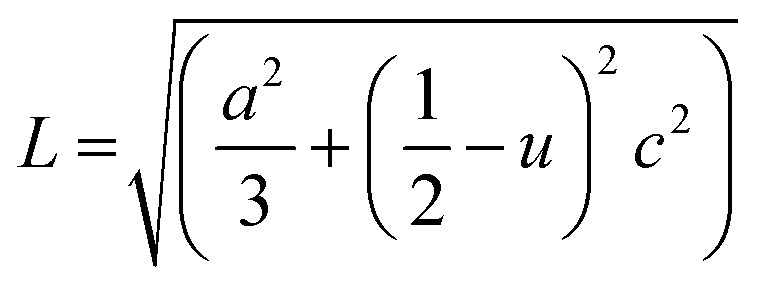

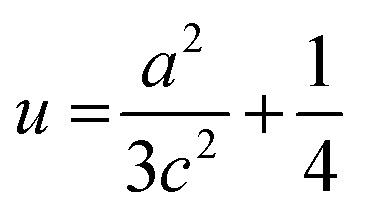
where *u* is the wurtzite structure parameter. *L* was found to be dopant dependent as listed in Table 1 and a distance from the bulk value 1.9767 Å of ZnO.^54^ This may be a result of the strain in the films.

**Fig. 2:**
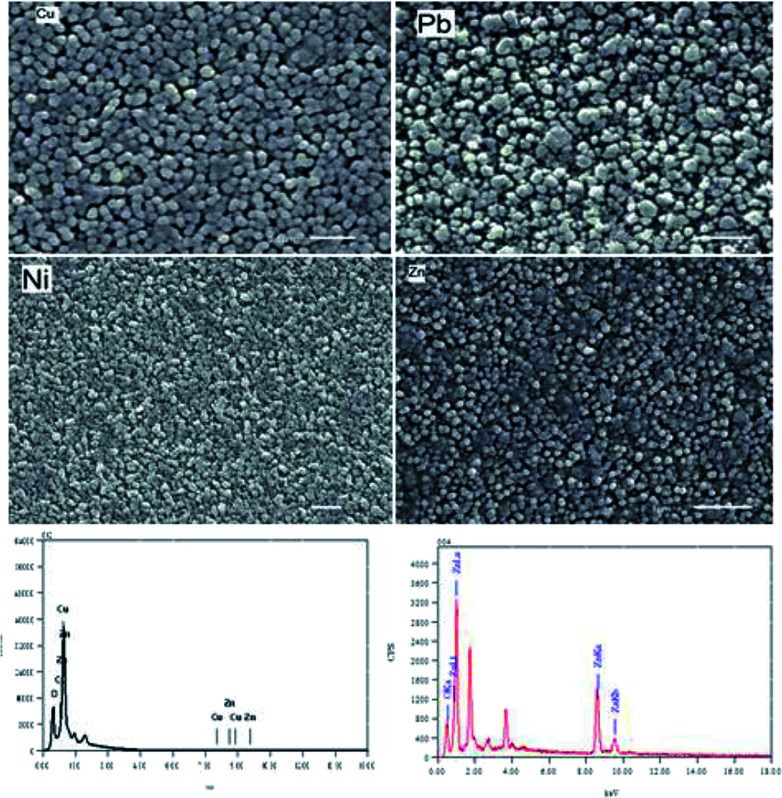
SEM images for all films and EDX for pure and Cu-doped films.

Fig. 2 shows the SEM images of the surfaces of pure and doped ZnO thin films. The average particle size has been calculated for all films using the cross section method. The average size was found to be 65.8, 102.75, 78.2 and 118 nm for pure, Cu, Ni and Pb doped ZnO thin films respectively. Comparing these results with the XRD values result we get a perfect match in the case of pure ZnO films. In the doped film cases higher sizes are measured from the SEM technique than XRD. The shift in the case of Ni-doped films is small but approximately doubles in size in the case of Cu and Pb doped films. That could be attributed to an agglomeration process take place during film formation. Circular grains are observed in pure and Cu-doped thin films. Different particles morphologies are noticed in the cases of Ni and Pb doped films. That could be attributed to the change in the growth mode with increasing the film thickness for the doped ZnO film. The EDX analysis shows the existence of the doped elements in all films. The atomic ratios of Cu, Ni and Pb percentage to Zn was found to be 10.95, 2.2 and 5.6% respectively.

**Fig. 3:**
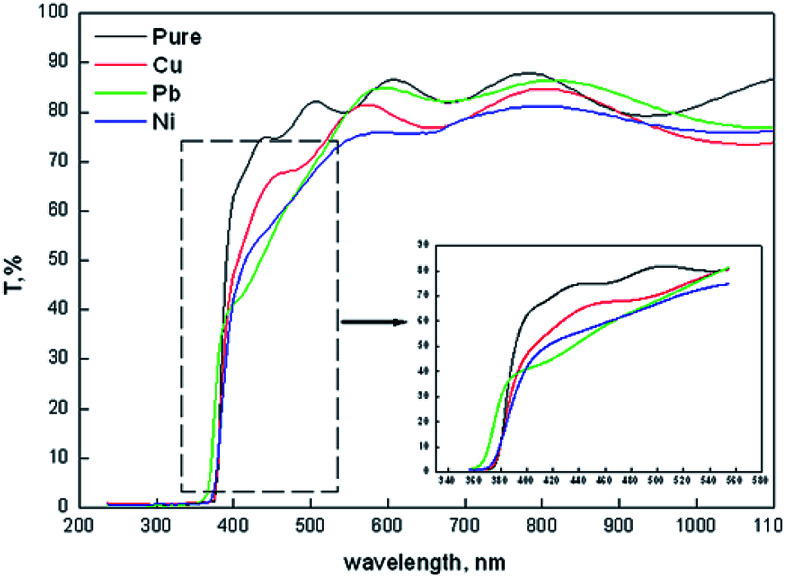
Optical transmission of pure and (Ni, Cu and Pb) doped ZnO thin films.

Fig. 3 shows the transmission spectra of all films in the spectral range of 200–1100 nm wavelength. All films show interference fringes which facilitate the thickness extraction from the transmission spectra using Swanepoel's method. As listed in Table 1 the thickness shows random variation against the type of doping. Generally it decreased upon doping. The Pb-doped film is thicker than Cu-doped films which in turn is thicker than the Ni-doped films. Such variation in the film thickness could be attributed to the reduction in the *c*-axis growth of all doped films with respect to the pure zinc oxide film. Also doping may cause a reduction in the reaction rate which slows down the film formation on the surface of the substrate. As an example of that, the bond length of Pb–O (2.49 Å (ref. 55)) is larger than Zn–O (1.97 Å (ref. 56)) bond length. This reflects that the energy of the Pb–O bond is lower than the Zn–O bond. This makes it easier for the oxygen attached to the Pb atom to escape to the film than the oxygen attached to Zn-ions. That will not help to bring more materials in the film bulk and help to decrease the film thickness. Also the generated strain in all films, compressive or tensile, leads to an increase in the strain energy which limits the dopant solubility in the host ZnO.^57^ The thickness of ZnO thin films shows a random variation upon doping as recorded by Bouznit *et al.*^58^

A general reduction in the transmission level can be observed upon ZnO doping. The average transmission of all film was found to decrease from 83.7, 78.61, 81.65 and 77.2 for pure and Cu, Pb and Ni doped ZnO films in the spectral range 500–1100 nm. The inset at the lower corner in Fig. 2 showed an enlarged part of the high absorption region in the transmission spectra in the range of 360–580 nm. Except for the Pb doped film, there is no shift in the cut off wavelength in transmission.

The thickness of all films was determined using the Prava program.^59^ The thickness values are listed in Table 1. As recorded, the thicknesses of all doped films were lower than the pure ZnO film.

Fig. 3 shows the plots of (*αhν*)^2^ against *hν* in order to estimate the band gap values of the deposited films. The band gap energy values *E*_g_ was estimated using the equation of Tauc's;^60^(*αhν*)^2^ = *A*(*hν* − *E*_g_)where *ν* is the photon frequency. The curve resulting from Tauc's equation has straight line portions and the band gap has been estimated by extrapolating the straight line to intersect the photon energy axes. The intersection point is the band gap value in eV. As estimated from those curves the band gap is almost constant for pure and doped (Cu, Ni) ZnO thin films at a band gap value of 3.2 eV. A small blue shift in this value is recorded for Pb doped ZnO films where the band gap value is observed at 3.3 eV.

In the case of Cu doping, the band gap was fund to be constant. That may be attributed to the low doping level which is not enough, in this case, to produce a notable shift in the band gap value of ZnO thin films even with the change in the degree of crystallinity which recorded. In the work of Jongnavakit *et al.*^45^ and Sreedhar *et al.*^61^ the band gap was found to increase with increasing Cu doping. This increase in the band gap has been attributed to the strong mismatch in the electronegativity of Cu and Zn atoms in ZnO. This effect may be compensated by the reduction in the degree of crystallinity found in our work. Reduction in crystallinity may introduce energy states in the band gap region which cause a reduction in the band gap value. The same result was recorded in ZnO thin films upon Ni doping in other work.

Ni-doping is known to decrease the band gap of ZnO thin films.^46,49^ The decrease in the band gap has been attributed to the strong sp–d exchange interaction between the band electrons and the localized ‘d’ electrons of the Ni ions substituted in the Zn sites in 10 at% Ni doping level.^46,62,63^

In our work the band gap was found to be constant in the case of Cu and Ni doping films while increasing in the Pb doped film. The constant band gap in the Cu and Ni doped films could be attributed to the low concentration of dopants which make no shift in the band gap value.^63^

On the other hand the optical band gap increases in the Pb doped films. Since the ionic radii of Pb^2+^ (1.29 Å) and Pb^4+^ (0.78 Å) are larger than the ionic radii of Zn^2+^ (0.74 Å), Zn^2+^ can be replaced by Pb^4+^ more easily than Pb^2+^ due to its close ionic radii. If Zn^2+^ ions are replaced by Pb^4+^ ions in the ZnO structure, Pb^4+^ ions will introduce two electrons into the ZnO, so it is expected to increase the charge carrier concentration and increase the oxygen content in the film. Such increase in the charge carrier concentration is the reason for the increase in the band gap upon Pb doping according to Moss–Burstein effect. Such increase in the band gap value upon Pb doping in ZnO thin films was recorded by Turgut *et al.*^53^

### Photoelectrochemical (PEC) properties

**Fig. 4:**
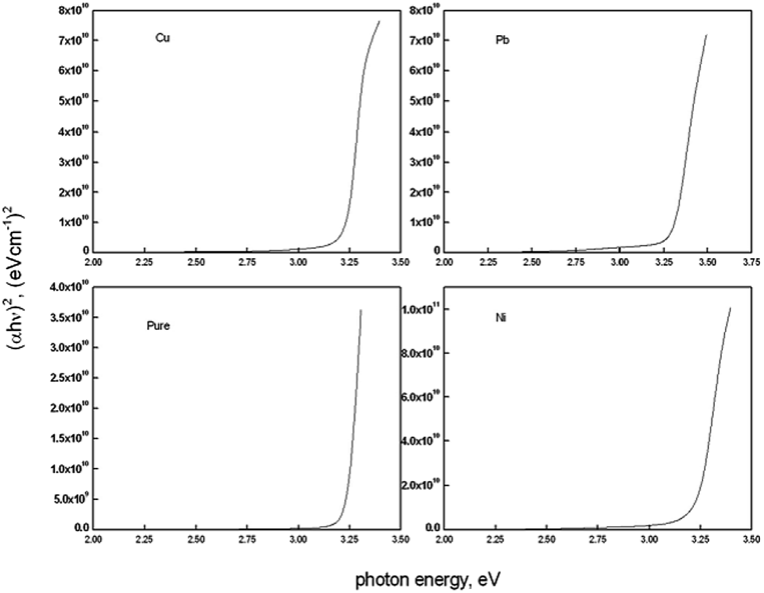
Plot of (*αhν*)^2^ against *hν* for all films.

The photoelectrochemical (PEC) behavior of the Ni, Cu, and Pb-doped ZnO electrodes were measured in dark and white light illumination as shown in Fig. 4(a–c). The highest obtained current density is 0.25, 5, and 8 mA cm^−2^@1 V for Ni, Cu, and Pb-doped ZnO, respectively. The Pb doping gave the highest photocurrent of the ZnO photoelectrodes. The photocurrent enhancement for Pb-doped ZnO can be ascribed to better charge separation, band gap shift and collection efficiency under white light exposure.

**Fig. 5:**
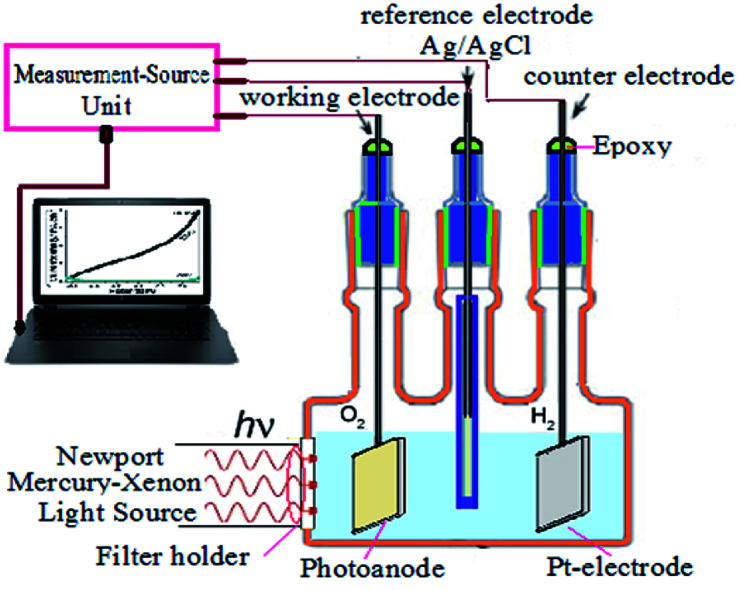
Schematic diagram of the used PEC water splitting system.

Fig. 5 shows the PEC behaviors of the Pb-doped ZnO electrode under white light illumination. As shown in Fig. 5(a), the power density was varied from 50 to 100 mW cm^−2^. As the power density increased, the current density is linearly increased with a rate of 61.2 mA W^−1^, Fig. 5(b). Upon exposure to light, the large surface area of the electrode will produce a high density of electron–hole pairs, which will motivate the splitting of H_2_O molecules under the effect of light to carry out the PEC reaction. From Fig. 5(a), the Pb-doped ZnO electrode is affected by the light and works as a photoanode. The current density values for an electrode at dark and light is 0.4 and 8 mA cm^−2^ at 1 V, respectively. This indicates an efficient electrochemical water splitting utilizing the Pb-doped ZnO electrode. The stability of the Pb-doped ZnO electrode for H_2_ generation was investigated as a function in the number of measurement runs and as a function of the illumination time. As shown in Fig. 5(a) and its inset, the current density-potential measurements were carried out for six successive runs at power density 100 mW cm^−2^. As the number of runs increased, the current density decreased from 8 to 7.22 mA cm^−2^ at 1 V. Then after 6 runs, the degradation rate of the photocurrent density for Pb-doped ZnO electrode was approximately 10%. Also, the stability of the Pb-doped ZnO electrode for H_2_ generation was investigated under white light illumination for a prolonged time as shown in Fig. 5(c). During this measurement, a small bias voltage of 0.6 V was applied between the photoanode and the counter electrode to overcome any external losses of the measuring system. The current density decreased sharply in the first period (130 s) from 8.88 to 0.29 mA cm^−2^, then almost constant till 1800 s, in which the accumulated ionic charges are increased, suggesting a longer lifetime of the suggested electrode to work in the PEC-water splitting experiment. It was reported that the high density of surface states might lead to a significant pinning of the Fermi level that can facilitate the participation of these defect states in the surface oxidation process, leading to a significant degradation of the photoanode.^64,65^

**Fig. 6:**
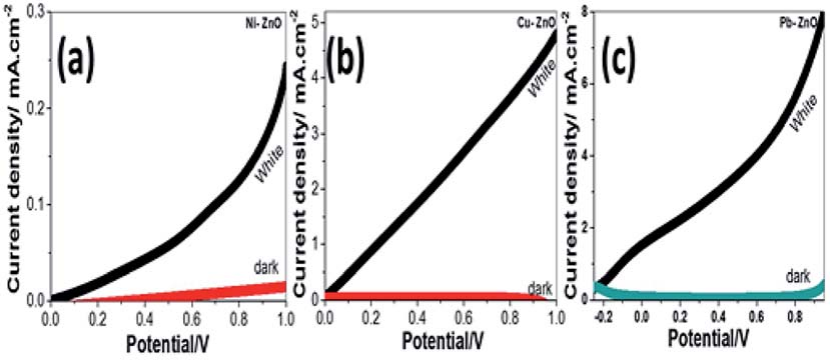
Photocurrent density-potential behavior for (a) Ni, (b) Cu, and (c) Pb-doped ZnO electrodes under dark and white light illumination at 298 K.

Fig. 6(a) shows the variation of photocurrent density with the potential from 0 to 0.65 V under the illumination of monochromatic light on Pb-doped ZnO electrode. The optical filters of different wavelengths from 390 to 636 nm are used to control the illumination at 25 °C. From the figure, the current density increases with increasing the incident wavelength from 390 to 460 nm; then it decreases as the wavelength increases from 460 to 636 nm. The illumination with light of wavelength 460 nm showed the highest response, however the longer wavelength, 636 nm, showed the lowest response.

This behaviour matches with the optical UV-visible analyses data as shown in Fig. 2. The behaviour of the photoanode can be tentatively attributed to the enhanced solar absorption that can cover a large portion of the solar spectrum. The photostability of the Pb-doped ZnO photoanode was also investigated during PEC reaction under different monochromatic light illumination. Fig. 6(b) shows the variation of photocurrent density with the illumination time at 0.6 V under the illumination of monochromatic light of different wavelengths. It can be seen that, during PEC water splitting reaction, the photocurrent density increased for the first 900 s of irradiation before becoming stable. This increased current density can be attributed to the modification of the surface of the electrode; a similar phenomenon was previously reported for the different types of electrodes.^66–68^

**Fig. 7:**
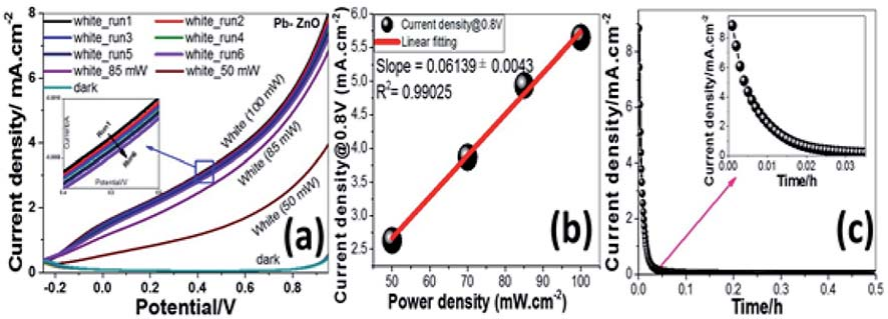
(a) Photocurrent density-potential curves at different power densities under white light illumination and (b) Current density versus power density of the incident light, and (c)Photocurrent density-time behavior at 0.6V under white light illumination.

The enhanced solar absorption of the Pb-doped ZnO photoanode was further confirmed by measuring the incident photon-to-current conversion efficiency (IPCE) under monochromatic illumination conditions. Such analytical measurements can also give meaningful insight into the contribution of Pb-doped ZnO electrode in the conversion of the incident photons into charge carriers. The IPCE was determined at an applied potential of 0.5 V from eqn (1) and shown in Fig. 7(a):^68,69^1
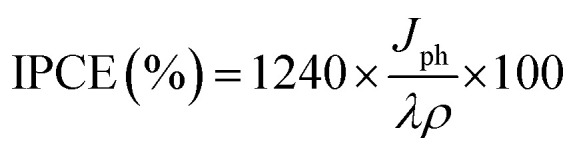
where *λ* is the wavelength of the illuminating monochromatic photons, *J*_ph_ is the photocurrent density, and *ρ* is the illuminating light power. The value of the IPCE decreased from ∼30% to 3% with increasing the wavelength from 390 to 636 nm.

**Fig. 8:**
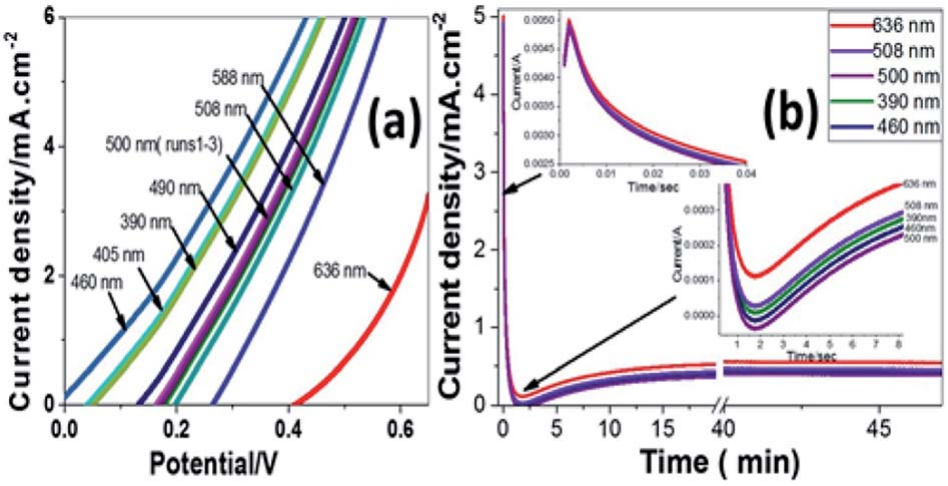
(a) Photocurrent density-potential and (b) photocurrent density–time characteristics under different monochromatic light illumination at 298 K.

To fully evaluate the PEC-water splitting performance of the Pb-doped ZnO photoanode, we further measured the applied bias photon-to-current efficiency (ABPE), which can allow diagnostic measurements represent the development of the electrode performance as a function of the applied potential. The measured ABPE values for the Pb-doped ZnO photoanode was calculated by using eqn (2): ^68,70^2
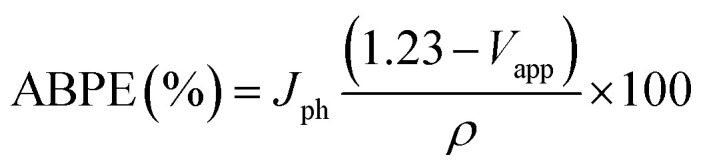
where *J*_ph_ is the measured photocurrent density, 1.23 is the standard state reversible potential of water, |*V*_app_| is the applied potential during the measurement of the photocurrent density, and *ρ* is the illuminating light power density. As displayed in Fig. 7(b), as the applied potential increased, the ABPE efficiency attained its maximum value and then decreased again when the applied potential was approaching the thermodynamic WS potential (1.23 volt). The maximum efficiency (ABPE = 0.6355) was obtained at 0.5 V for this electrode (Fig. 8).

## 4. Conclusion

3 wt% Cu, Ni, and Pb-doped ZnO thin films have been deposited using a low-cost spray pyrolysis technique. The effect of metallic (Cu, Ni, and Pb) doping on the photoelectron/chemical properties of zinc oxide thin films was studied. The structural information of the prepared films has been extracted addressed using X-ray diffraction, whereas the grain sizes of all doped films have the same value of 50 nm. The average transmission was found to decrease from 83.70% for pure ZnO film to 81.65%, 78.61%, and 77.2% for Pb, Cu, and Ni-doped ZnO films. Also, the energy band was blue shifted from 3.2 eV for pure to 3.3 eV for the Pb-doped ZnO film. The PEC behavior of Ni, Cu, and Pb-doped ZnO photoelectrodes were applied for solar H_2_ generation from H_2_O. The Pb-doped ZnO showed the highest photocurrent under white light and monochromatic illumination in 0.3 M Na_2_SO_3_ electrolyte solution. The Pb-doped ZnO photoelectrode showed high stability in terms of the number of H_2_ production runs and exposure time. Finally, the optimized incident photon-to-current conversion efficiency and applied bias photon-to-current efficiency were ∼30% at 390 nm and 0.636 at 0.5 V, respectively. Finally, the reported efficiencies values in this study are higher than that previously reported for different ZnO-based photoelectrodes.

## Conflicts of interest

There are no conflicts to declare.

## Supplementary Material
